# Connectivity modelling in conservation science: a comparative evaluation

**DOI:** 10.1038/s41598-022-20370-w

**Published:** 2022-10-06

**Authors:** Siddharth Unnithan Kumar, Samuel A. Cushman

**Affiliations:** 1 Mathematical Institute, University of Oxford, Oxford, United Kingdom; 2 Wildlife Conservation Research Unit (WildCRU), Department of Zoology, University of Oxford, Oxford, United Kingdom; 3 US Forest Service, Rocky Mountain Research Station, Flagstaff, AZ USA

**Keywords:** Conservation biology, Ecological modelling

## Abstract

Landscape connectivity, the extent to which a landscape facilitates the flow of ecological processes such as organism movement, has grown to become a central focus of applied ecology and conservation science. Several computational algorithms have been developed to understand and map connectivity, and many studies have validated their predictions using empirical data. Yet at present, there is no published comparative analysis which uses a comprehensive simulation framework to measure the accuracy and performance of the dominant methods in connectivity modelling. Given the widespread usage of such models in spatial ecology and conservation science, a thorough evaluation of their predictive abilities using simulation techniques is essential for guiding their appropriate and effective application across different contexts. In this paper, we address this by using the individual-based movement model Pathwalker to simulate different connectivity scenarios generated from a wide range of possible movement behaviours and spatial complexities. With this simulated data, we test the predictive abilities of three major connectivity models: factorial least-cost paths, resistant kernels, and Circuitscape. Our study shows the latter two of these three models to consistently perform most accurately in nearly all cases, with their abilities varying substantially in different contexts. For the majority of conservation applications, we infer resistant kernels to be the most appropriate model, except for when the movement is strongly directed towards a known location. We conclude this paper with a review and interdisciplinary discussion of the current limitations and possible future developments of connectivity modelling.

## Introduction

Your task is not to seek for love, but merely to seek and find all the barriers within yourself that you have built against it. *- Jalaluddin Rumi*

### Landscape connectivity

Movement is fundamental to all ecological processes in our more-than-human world^1–3^. In conservation science, it is commonly studied in the context of gene flow, dispersal, population dynamics, and shifting habitats in response to changes in climate and human activity^4–6^. Here, animal movement is often understood in response to a range of biotic and abiotic environmental factors, constituting a complex relationship between individuals and the landscape that is fluid in space and time, and which manifests at different scales^7–9^. Understanding and predicting the pathways of organism movement thus forms a cornerstone of ecological science and its application to conservation practice. However, it is usually very challenging to model accurately due to the countless factors which can shape and affect movement patterns^10^.

‘Landscape connectivity’, commonly defined to be the extent to which a landscape facilitates organism movement, is an emergent and dynamic phenomenon based on the cumulative movement pathways of individuals across time and space^11^. Conceptually, it provides a tractable and powerful methodology for analysing and mapping organism movement patterns, and its widespread utility has been established across an enormous body of ecological work^12,13^. As such, the theory, modelling and prediction of landscape connectivity has grown to become a central focus of applied ecology and conservation science^14^.

### Modelling connectivity

#### Resistance surfaces

Several techniques for modelling landscape connectivity have been developed in recent years^15^. The most prominent methods today use ‘resistance surfaces’, which are pixelated maps (in the form of geospatial image layers) that provide the spatially-explicit input data necessary for modern connectivity models^16^. Each pixel of the surface is assigned a numerical value which reflects the estimated ‘cost of movement’ through the region of the landscape corresponding to that pixel, giving a spatiotemporally static approximation to how landscape structure affects movement.

Using these resistance surfaces, connectivity models typically then evaluate landscape connectivity with an algorithm based on either cost-distance measurements, or electrical circuit theory from classical physics^14^. Despite its widespread usage as the basis for modern connectivity models, the framework of landscape resistance does have its limitations as a basis for modelling animal movement, which we will revisit in the discussion section.

#### Connectivity algorithms

The simplest cost-distance algorithm is the ‘least-cost path’, which identifies a path (or corridor) between two geographical locations on the resistance surface that minimises the accumulated cost of movement between those locations^17^. This was later extended by synoptic approaches like the factorial least-cost path algorithm, which computes the least-cost paths between any set of source points simultaneously^18^.

However, there are severe limitations to these least-cost path approaches in practice^19^. Centrally, there is little reason to assume that an animal knows (or even thinks in terms of) the route of the least-cost path. Moreover, the destination may not be known to the animal; even if this were so, obtaining the knowledge of their precise destination can be very difficult, especially with dispersing animals. To remedy this latter issue, the resistant kernels method was developed^20^. It is a cost-distance algorithm that estimates connectivity as a function of source locations, landscape resistance and dispersal thresholds, without requiring knowledge of destination points.

**Figure 1:**
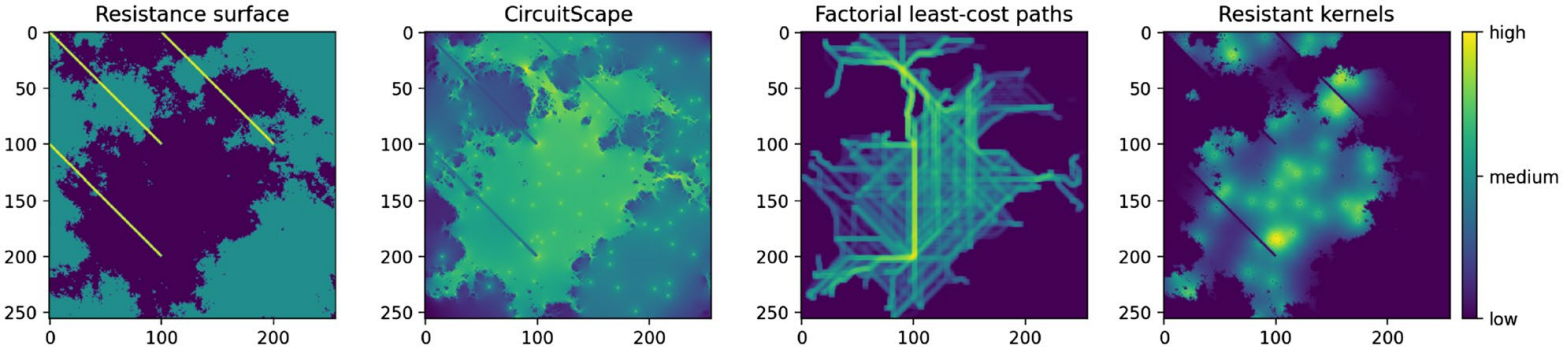
An example of a resistance surface (left), and the resulting connectivity predictions of Circuitscape, factorial least-cost paths and resistant kernels applied to this resistance surface. The input data for these models are the resistance surface, together with 100 uniformly randomly selected source points on the resistance surface.

In contrast, based on electrical circuit theory, Circuitscape is a connectivity model which simulates electrical current flow across a resistance surface^21^. Source locations of movement are treated as nodes of a circuit, with resistance values as the strength of electrical resistors, and animals modelled as electrons ‘flowing through this circuit’. This algorithm produces a current density for each pixel on the resistance surface, with higher current values assumed to reflect higher degrees of connectivity. See Fig. 1 for an illustration of the outputs of Circuitscape, factorial least-cost paths and resistant kernels.

### Using simulation to evaluate connectivity model performance

#### Simulated versus empirical data

Simulation experiments are essential for comparing the performance of these computational models. At present, there exist a wide range of studies using empirical data which evaluate the abilities of connectivity models to match observed movement patterns^22–25^. These analyses typically investigate model performance by measuring the degree of correlation between empirical movement data and the predictions from different connectivity algorithms. However, in an empirical analysis, the relationships driving the observed movement remain unknown. Moreover, the model predictions are dependent on several factors which are also not empirically known, such as: dispersal ability, spatial scale of movement choice, mortality risk, density and distribution of the studied population, and how landscape structure affects movement.

As a result, the degree to which a connectivity model prediction matches observed movement paths does not necessarily demonstrate the model’s accuracy relative to other methods. This is because the many uncontrolled factors in an empirical study mean that *the relationships driving movement patterns need not be reflected in the data*. Thus, although empirical connectivity analyses can give tremendous insight in many contexts, they are limited in their ability to comparatively evaluate the performance of different connectivity models. Working instead with suitably generated simulated data allows us to compare model predictions with a ‘known truth’ - namely, the connectivity maps generated by simulated movement paths resulting from a controlled set of known parameters - and thus enables a more definitive analysis of model performance.

#### The utility of simulation experiments

Simulation frameworks have been instrumental for testing key hypotheses in many branches of spatial ecology. For example, in landscape genetics, simulated data generated by models such as CDPOP have been utilised to study central questions in this field which cannot be addressed with empirical data alone^26–29^. Simulation techniques have also recently been used in species-specific landscape connectivity studies, such as with the Sunda clouded leopard^30^ and northern spotted owls^31^. Yet at present, there is no published comparative evaluation which utilises a simulation framework to measure the relative performance of the connectivity models themselves. Given the widespread usage of Circuitscape, resistant kernels and factorial least-cost paths across an enormous range of connectivity analyses in recent years, a rigorous evaluation of their predictive abilities using simulation techniques is essential for guiding the appropriate and effective application of these connectivity models in conservation science.

In this paper, we used simulated data from the movement model Pathwalker (described below, see also^32^) to address this fundamental question in the field of connectivity modelling. Namely, how accurate are (1) factorial least-cost paths, (2) resistant kernels, and (3) Circuitscape, in predicting landscape connectivity as we vary movement behaviour and spatial complexity over a wide range of parameters? Pathwalker is an individual- and process-based, spatially-explicit movement model which simulates organism movement on a resistance surface as a function of several parameters, including: energetic cost of movement, landscape resistance, mortality risk, autocorrelated movement, and bias towards a known destination, all at multiple spatial scales.

The flexibility and detail of Pathwalker enable us to test a variety of hypotheses concerning the patterns of landscape connectivity which result from differing movement choices, allowing for a comprehensive comparison of major connectivity models used in conservation science. This means that we are able to quantify the extent to which resistant kernels, Circuitscape and factorial least-cost paths can approximate connectivity pathways through a landscape, giving an in-depth picture of their relative abilities in a wide range of contexts. The simulated data from Pathwalker is generated by stochastic movement rules which reflect and extend the assumptions that these three connectivity algorithms use to model movement behaviour, making it a suitable framework with which to analyse their predictive abilities.. Furthermore, with its ability to simulate movement pathways as a function of ecological processes in much greater detail than other mainstream connectivity models, Pathwalker itself provides a powerful new tool for understanding and mapping landscape connectivity.

## Methods

### Creating the connectivity model predictions

**Figure 2:**
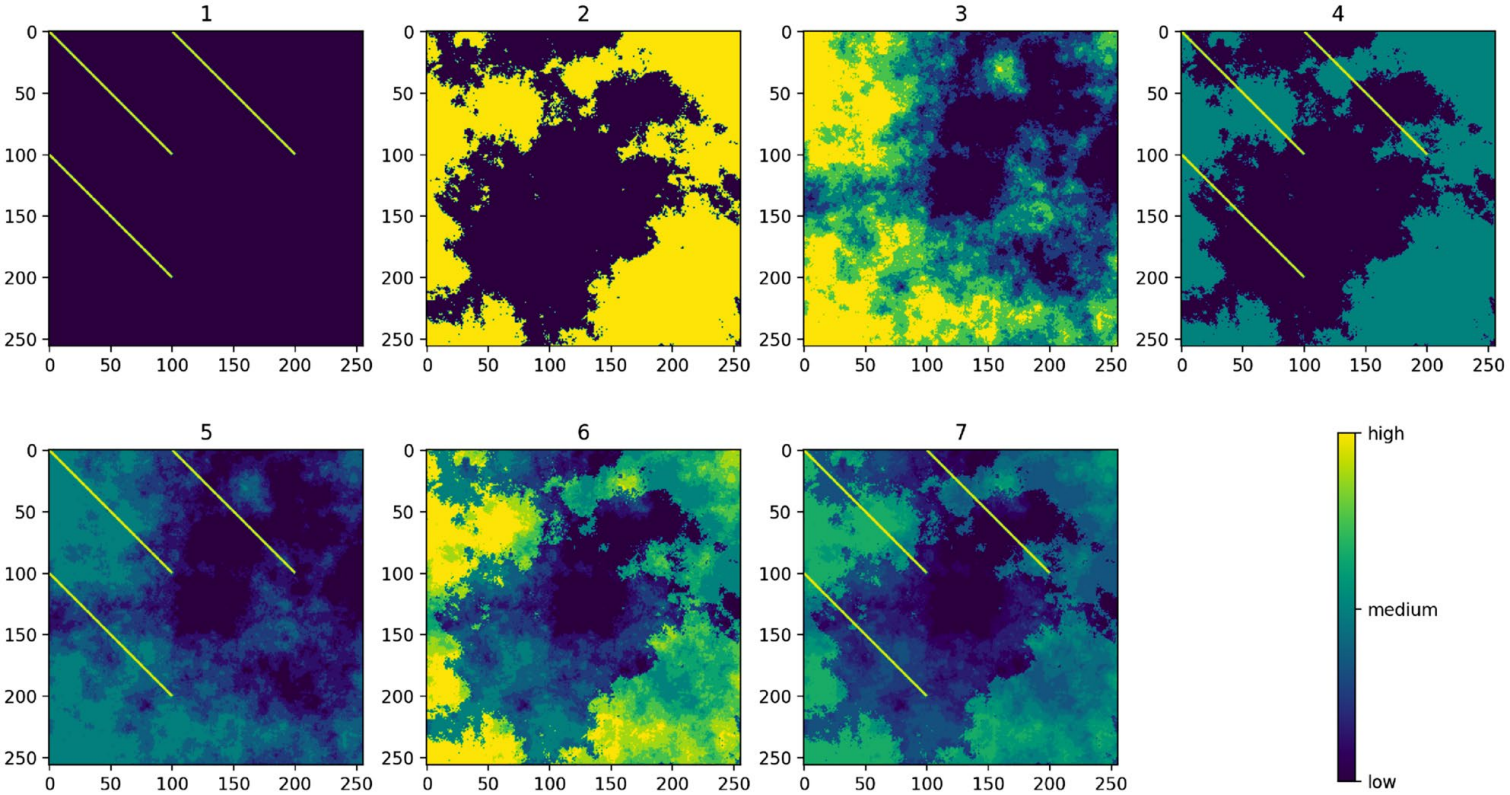
The seven resistance surfaces used in the analysis.

We begin by simulating 7 resistance surfaces of 256x256 pixels, which increase in complexity from a simple uniform landscape with three barriers, to surfaces with more continuous and varied simulated landscape features (Fig. 2). We also randomly select 100 points in a 256x256 grid, which act as the starting locations for the movement on the resistance surface. Each of these 7 surfaces, together with the 100 source points, is then given to our three connectivity models: factorial least-cost paths, resistant kernels and Circuitscape. For each of the 7 resistance surfaces, we thus obtain three predictions for landscape connectivity, one from each model. In our analysis described below, we test which of these three models best matches the actual connectivity pathways generated from the movement data simulated by Pathwalker, across a wide variety of scenarios arising from different simulated movement behaviours. All analyses in this study are performed using Python version 3.8 (https://www.python.org) with the SciPy package^33^, except for the redundancy analysis and variance partitioning, which are performed using R version 3.6 (https://www.r-project.org) with the vegan package^34^.

### Pathwalker

Pathwalker simulates individual organism movement from a source point on a resistance surface as a biased random walk, which is a function of three basic movement mechanisms - energy, attraction and risk. These mechanisms can be used individually, or together in pairwise or three-way combination. The energy mechanism simulates the dispersal and energetic capabilities of an organism, represented by the energetic cost of movement across the resistance surface, and produces an unbiased random walk which ends once the specified energetic cost threshold has been reached. The attraction mechanism produces a random walk in which the movement is biased towards pixels of lower resistance values, resulting in a spatially-determined movement choice. The risk mechanism simulates mortality risk during movement by producing an unbiased random walk on a ‘risk surface’ (which can be proportional to the resistance surface, or an entirely different surface); the walk can probabilistically end at each step, with a higher likelihood of ending on pixels with higher risk values.

**Figure 3:**
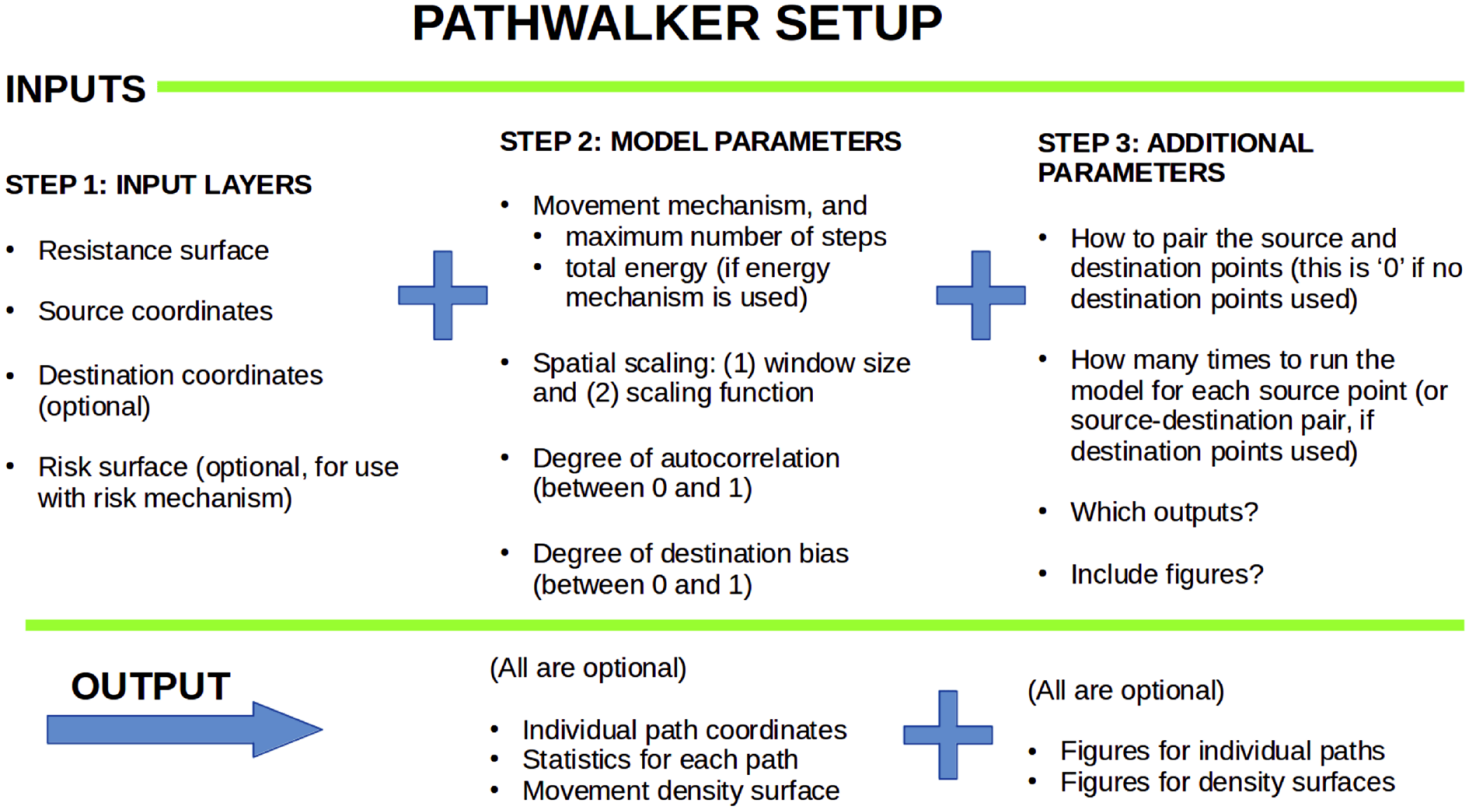
Schematic for configuring the Pathwalker movement model.

The movement mechanisms are compatible with a multiple-scale response to the spatially heterogeneous resistance surface: the energy, resistance and risk values can be calculated using the mean, maximum or minimum value of a focal window of size *n* around each pixel. Additionally, there are two directionality parameters: an autocorrelation parameter *C*, which determines the likelihood of continuing in the current direction of movement; and a destination bias parameter *D*, which governs the extent to which the walk is biased towards a particular destination on the resistance surface. This is summarised by the schematic in Fig. 3, but see^32^ for more details on Pathwalker.

### Creating the simulated connectivity maps

**Figure 4:**
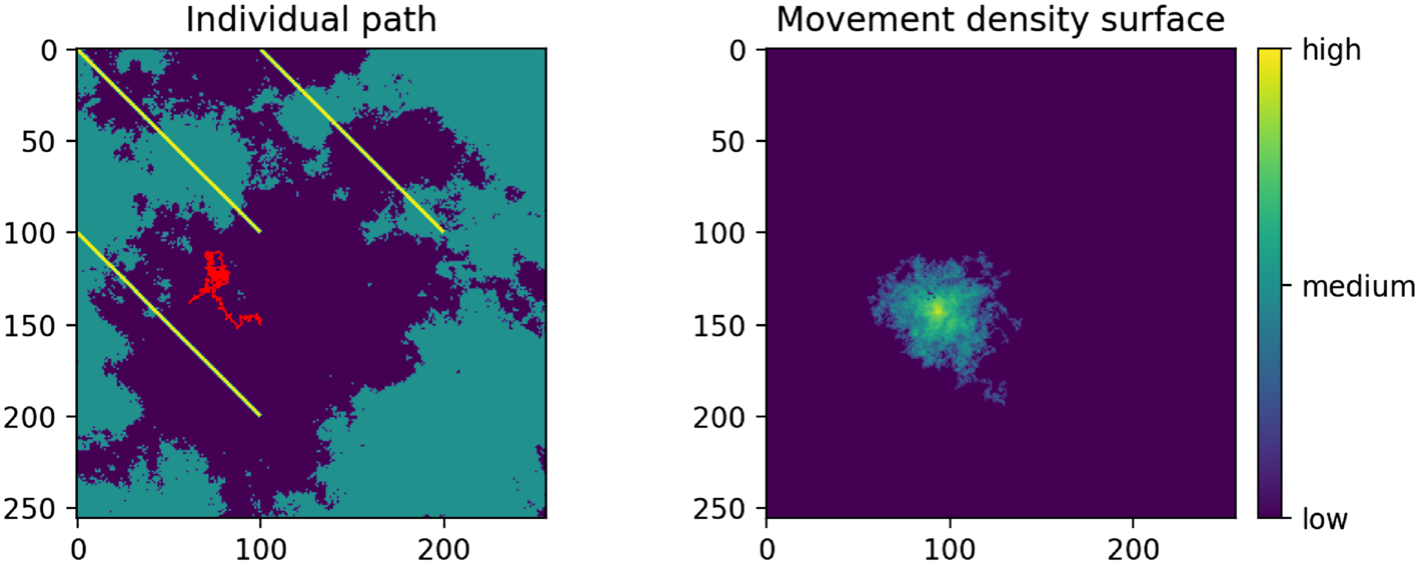
On the left, an example individual stochastic path in red on the fourth resistance surface used in this analysis; yellow is high resistance and blue is low resistance. On the right, an example density surface produced by aggregating 100 paths from the same source point and with the same movement parameters; yellow is high density and blue is low density.

Pathwalker outputs individual movement paths generated by these parameter combinations. It can then aggregate these movement paths into a ‘density of movement’ surface, which provides the simulated connectivity surface to which we compare the three connectivity model outputs. Figure 4 shows an example density surface, produced by aggregating 100 paths run from a single source point on a resistance surface. In our analysis, for each parameter configuration (which represents one possible combination of movement behaviours), we run Pathwalker 100 times from each of the 100 randomly selected source points; we then aggregate these 10,000 movement paths to create a single connectivity surface which results from the movement choices of this particular set of behavioural parameters. The length of each path in the analysis is capped at 500 steps.

In order to provide a comprehensive comparison of factorial least-cost paths, resistant kernels and Circuitscape, we perform two in-depth analyses. First, we test their accuracy with each of the 7 resistance surfaces across 252 possible configurations of movement behaviour, sampling the full parameter space of movement mechanism, spatial scaling and degree of autocorrelation, but without including destination bias. Explicitly, on each of the 7 resistance surfaces, we run 7 different movement mechanisms, 4 degrees of spatial scale, 3 types of scale response, and 3 degrees of autocorrelation, giving a total of 1,764 simulated connectivity scenarios (Table 1).

For a second analysis, we then test the effect of 3 degrees of destination bias on the model accuracies, by varying this parameter together with the 7 different movement mechanisms while keeping the spatial scale, scaling function and autocorrelation fixed, providing an additional 21 movement scenarios for each of the 7 resistance surfaces (Table 1). This gives a further 147 connectivity scenarios, resulting overall in 1,911 simulated connectivity surfaces with which to compare the predictive abilities of factorial least-cost paths, resistant kernels and Circuitscape.

**Table 1:** The selection of parameters used to produce the simulated connectivity maps with Pathwalker. In both analyses, all 7 resistance surfaces are tested, and all 7 movement combinations are used (energy, attraction, risk, and their pairwise and three-way combinations). In the first analysis, the spatial scale, scaling function and degree of autocorrelation are varied; in the second analysis, these three parameters are fixed whilst the degree of destination bias is varied.

	First analysis	Second analysis
Resistance surface	All 7 resistance surfaces	All 7 resistance surfaces
Movement mechanism	All 7 movement combinations	All 7 movement combinations
Spatial scale	$1\times 1$, $3\times 3$, $5 \times 5$, $7 \times 7$	$1\times 1$
Scaling function	All 3 scaling functions	Focal mean
Autocorrelation	0, 0.35, 0.75	0.2
Destination bias	0	0.1, 0.3, 0.6
Total combinations	1764	147

### Measuring the accuracy of the connectivity model predictions

To measure the accuracy of these connectivity models in each of the 1,911 scenarios, we use three statistics: (1) the root-mean-squared error between the true and predicted surface, (2) the Pearson linear correlation coefficient between the true and predicted surface, and (3) the degree of spatial overlap between the areas of 10% highest connectivity in the true and predicted surface. For (1), we divide the total error by the number of pixels on the resistance surface, in order to obtain a scaled value between 0 and 1.

We subsequently perform a variance partitioning and a set of canonical correspondence analyses (CCA) on these three statistics, supported by appropriate boxplots and an analysis of variance. We do this first for the 1,764 surfaces which do not include movement biased towards a destination, and then separately for the 147 surfaces which do incorporate destination bias. These multivariate analyses allow us to synthesise our results over the whole collection of different simulated movement behaviours, and thus provide a definitive conclusion to the model accuracies across these behavioural scenarios.

### Summary of analysis methodology

To summarise, we first simulate 7 different resistance surfaces, together with 100 source locations on these surfaces. We run the three connectivity models - factorial least-cost paths, resistant kernels and Circuitscape - on these 7 surfaces, giving three different connectivity model predictions for each resistance surface.

For each resistance surface, we then stipulate a range of possible movement behaviours, which sample the full parameter space of mechanisms, spatial scales and degrees of autocorrelation; we use the Pathwalker software to simulate one connectivity map from each parameter configuration, giving us 1,764 simulated connectivity surfaces. Additionally, we test the effect of destination bias on model performance by varying this parameter together with the movement mechanisms for each resistance surface, while fixing the scaling and autocorrelation parameters, generating a further 147 connectivity surfaces.

We then measure how closely the three connectivity models compare with each of the 1,911 simulated connectivity maps, by calculating the average error, linear correlation and degree of spatial overlap between the true (simulated) and predicted surfaces in each case.

Finally, we synthesise these statistics using variance partitioning, canonical correspondence analyses and analysis of variance, supported by appropriate boxplots. From this we obtain a comprehensive picture for the accuracy of each of the three models across the possible movement behaviour parameters and resistance surfaces.

## Results

### Connectivity model performance without destination bias

**Figure 5:**
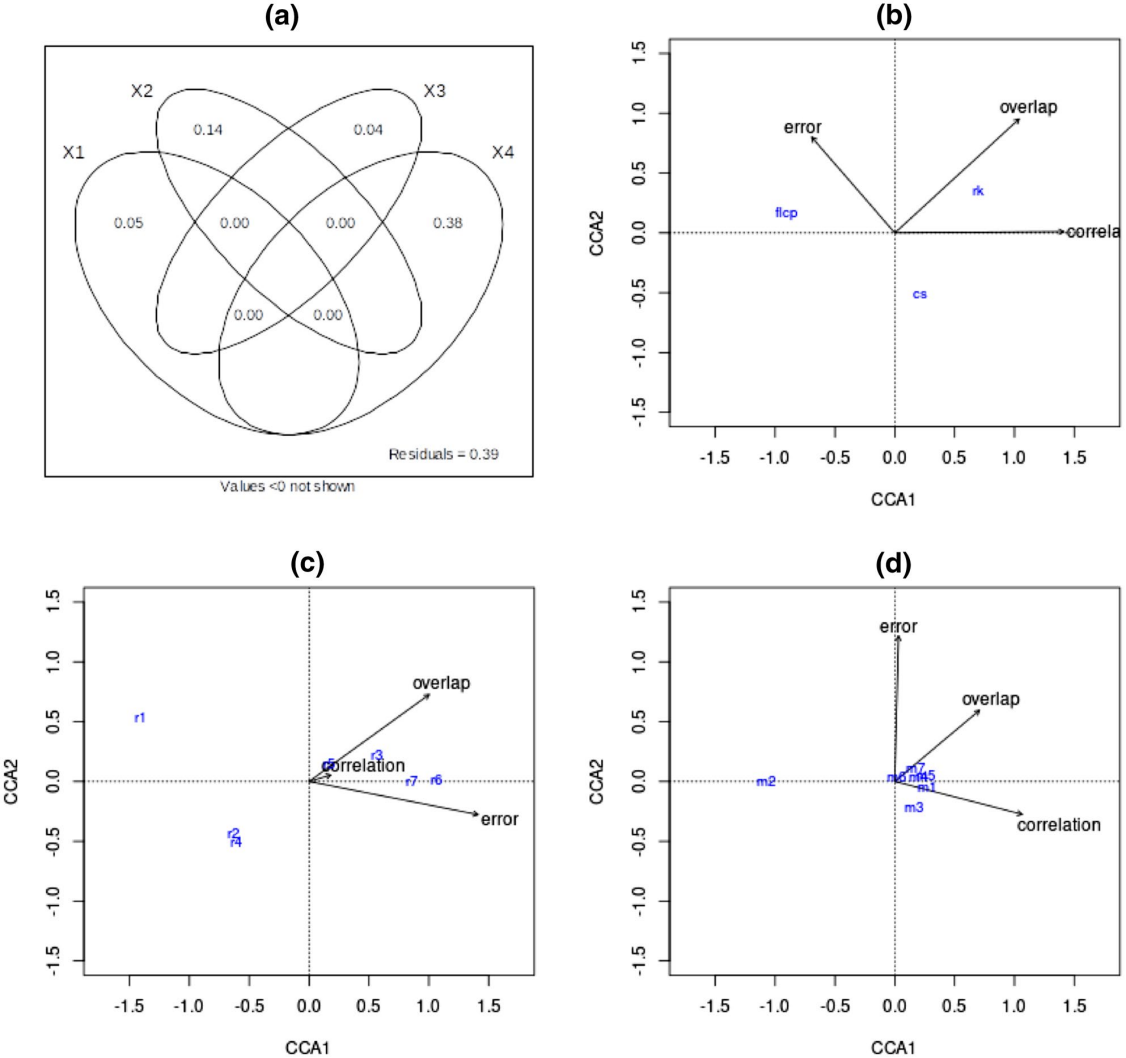
Variance partitioning (**a**) and canonical correspondence analyses (**b**–**d**) for the first investigation. A key to the CCA figure labels is provided in Supplementary Table S2. The variance partitioning shows model choice (X4) to be the major factor affecting predictive accuracy, more than twice the strength of the effects of spatial complexity of the resistance surface (X2), which in turn is roughly triple that of the mechanism (X1) and the remaining parameters - autocorrelation, scale and scaling function - combined (X3). In (**b**) we see that predictions by resistant kernels overall give the highest correlation and overlap, and those by Circuitscape result in the least error, whereas factorial least-cost paths is seen to be the least accurate with all three statistics. (**c**) shows that higher levels of spatial complexity result in great model error and overlap, with little effect on correlation. (**d**) demonstrates model performance to be much lower when using only the attraction mechanism, without any inclusion of energetic cost or mortality risk.

Recall that for the first analysis, across each resistance surface, we simultaneously vary the movement mechanism, autocorrelation, spatial scale and scale response function, without the movement being biased towards a known destination. The variance partitioning in Fig. 5a shows that the dominant factor in the accuracy of predicting connectivity is the choice of model used. More specifically, Fig. 5b shows that predictions using Circuitscape result in the least root-mean-squared error between the predicted and simulated connectivity maps, whereas the factorial least-cost paths model is the least accurate in all three statistics. Resistant kernels are seen to be the most accurate method overall, based on the linear correlation and spatial overlap between the predicted and true connectivity maps. This is shown numerically in Table 2.

Figure 5c demonstrates that variation in landscape structure can have a substantial effect on model performance: on average, as spatial heterogeneity increases, the models display much higher error but also substantially higher overlap, with little effect seen on the degree of linear correlation. Moreover, the four-way analysis of variance (Supplementary Table S1) shows very strong interaction between model accuracy and the choice of resistance surface. The boxplots in Supplementary Figure S2 show that the difference in model performance is much greater at lower levels of spatial complexity. As the spatial heterogeneity becomes more complex, on average, all three models display higher error; simultaneously, the correlation and overlap of factorial least-cost paths and Circuitscape increases, while that of resistant kernels remains consistently high across the variation in spatial complexity.

Figure 5d shows that these connectivity models are overall much less accurate if the movement is influenced only by landscape resistance values (which is parameterised by the attraction mechanism), without any limit on dispersal capabilities or mortality risk. This is seen by the position of the attraction mechanism (m2). More detail on the effects of movement mechanism on model performance is illustrated by the boxplots in Supplementary Figure S3. Specifically, with the presence of energy or risk, resistant kernels has much higher correlation and overlap than Circuitscape, and similar levels of error; without any threshold for dispersal or mortality risk, Circuitscape has substantially lower error, and similar levels of correlation and overlap.

Comparatively, the variance partitioning suggests that the effects of spatial scaling and autocorrelation are not seen to be major factors affecting the accuracy of the three connectivity models, as shown in the additional CCA diagrams in Supplementary Figure S1.

**Table 2:** Results from the first study, in which we test the accuracy of factorial least-cost paths, resistant kernels and Circuitscape across 1,764 connectivity scenarios without destination bias. This table displays the overall (1) average root-mean-squared error, (2) linear correlation and (3) degree of spatial overlap, between the simulated connectivity surfaces and the predictions given by these three models.

Method	Average error	Linear correlation	Spatial overlap
Factorial least-cost paths	0.0095	0.3943	0.3897
Resistant kernels	0.0071	0.6736	0.6070
Circuitscape	0.0062	0.5876	0.4503

### Connectivity model performance with destination bias

**Figure 6:**
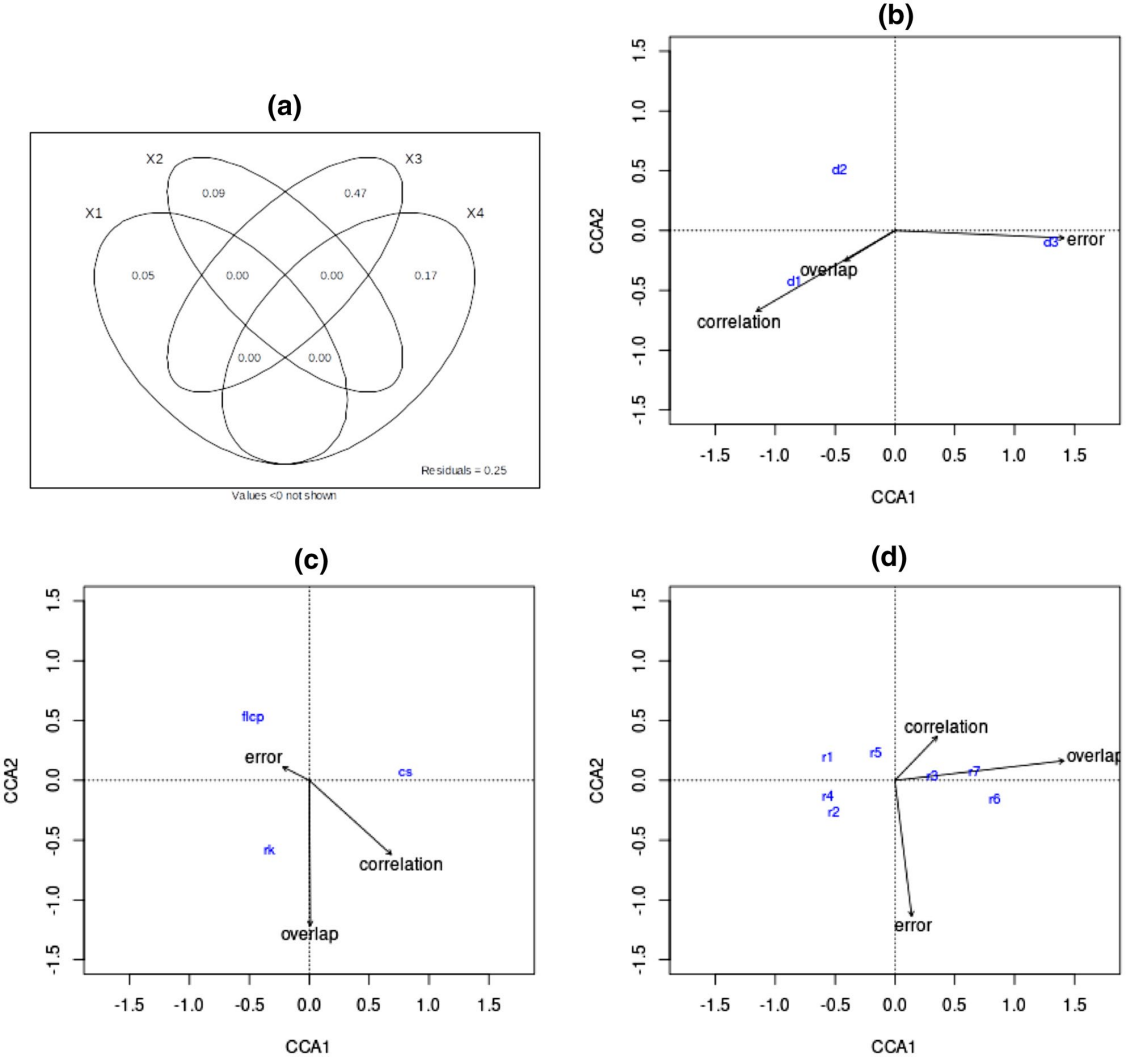
Variance partitioning (**a**) and canonical correspondence analyses (**b**–**d**) for the second investigation. A key to the CCA figure labels is provided in Supplementary Table S2. The variance partitioning now shows destination bias to be the primary factor determining the accuracy of connectivity predictions, approximately thrice as influential as model choice (X4), which in turn has a much greater effect on predictive accuracy than movement mechanism (X1) and spatial complexity (X2). In (**b**), we see that a lower degree of destination bias gives predictions which are more accurate across all three statistics. In (**c**) we find that predictions by resistant kernels overall give the highest overlap, and those by Circuitscape result in the least error, whereas factorial least-cost paths is seen to be the least accurate. (**d**) suggests that, with destination bias, higher spatial complexity now results in more accurate model predictions, with an increase in overlap and minor increase in correlation as spatial variation increases.

In the second study, we measure model accuracy when the movement is biased to differing degrees towards a known destination, and we vary the mechanisms and resistance surfaces but keep fixed the autocorrelation, spatial scale and scale response function. For this analysis, the variance partitioning in Fig. 6a shows destination bias to be the main driver in variation of model accuracy across the different connectivity scenarios, approximately three times as influential as the choice of model used. Figure 6(b) illustrates a very clear trend in model accuracy: as the degree of destination bias increases, the error increases and the correlation and overlap decrease.

In Fig. 6c, we see factorial least-cost paths to still be the least accurate by all three metrics. And as before, resistant kernels display the highest spatial overlap, and Circuitscape the lowest average error. This trend is in fact consistent over all degrees of destination bias, as seen with the boxplot diagrams in Supplementary Figure S4(a) and S4(c). However, the boxplot diagram in Supplementary Figure S4(b) shows that, for higher levels of destination bias, the connectivity predictions by Circuitscape produce the strongest linear correlations with the simulated connectivity maps; this results in the overall higher correlation seen with Circuitscape in Table 3. And in comparison with the first study, the presence of destination bias results in a stronger link between higher spatial complexity and greater model accuracy, as inferred from Fig. 6d.

**Table 3:** Overall results from the second analysis, which tested the accuracy of factorial least-cost paths, resistant kernels and Circuitscape across 147 connectivity scenarios.

Method	Average error	Linear correlation	Spatial overlap
Factorial least-cost paths	0.0150	0.3318	0.4160
Resistant kernels	0.0139	0.4627	0.5675
Circuitscape	0.0127	0.5276	0.4801

## Discussion

The theory, modelling and prediction of landscape connectivity has grown to become a central focus of applied ecology and conservation science, with widespread application in practice and policy. This has led to the development of sophisticated computational algorithms to estimate and map connectivity, with Circuitscape, resistant kernels and factorial least-cost amongst the most widely used. Despite the influential usage of these three models, there is at present no published analysis which comprehensively evaluates their relative predictive abilities with simulated data. Although many studies to date have tested and validated their predictions using empirical data, such analyses are limited in their ability to comparatively evaluate the accuracy and performance of these connectivity models, due to the unknown variables and relationships driving the movement patterns in empirical data.

In this paper, we have used the spatially-explicit individual-based movement model Pathwalker to generate different connectivity scenarios from a broad range of simulated movement behaviours and spatial complexities, and then measured the extent to which the three models are able to predict these simulated patterns of landscape connectivity. We are thus able to provide a clear and comprehensive evaluation of the performance of three of the most popular connectivity models in conservation science, giving crucial information for their effective use and application across different contexts.

### Analysis results

The results of our simulation analysis show particular patterns in model performance which remain constant across the various combinations of movement mechanism, spatial scaling, autocorrelation, destination bias and spatial complexity. Centrally, factorial least-cost paths proved the least accurate of the three models, being consistent in having the highest error and lowest correlation and spatial overlap. This demonstrates that, despite being a widely used approach to modelling connectivity, the precise least-cost path (or corridor) between two locations on a resistance surface may dramatically not reflect the actual pathways of animal movement.

We also find resistant kernels to consistently produce predictions which result in the greatest degree of spatial overlap with the simulated connectivity maps, substantially higher than both Circuitscape and factorial least-cost paths. In terms of the other two statistics measuring accuracy - root-mean-squared error and linear correlation - the comparative predictive abilities of resistant kernels and Circuitscape vary according to two factors: primarily, the degree to which movement is inclined towards a known destination; and to a lesser degree, the spatial complexity of the resistance surface. Other movement parameters in Pathwalker did not affect model performance to same the extent as destination bias and spatial heterogeneity.

Without destination bias, resistant kernels produced connectivity predictions which correlated and overlapped more strongly with the simulated connectivity scenarios than those given by Circuitscape, across all degrees of spatial complexity, suggesting resistant kernels to be the most robust model regarding variation in landscape structure. Moreover, between these two models, the outputs from resistant kernels resulted in a smaller error for lower degrees of spatial complexity, with the reverse being true for higher spatial complexity. However, when the movement was strongly biased towards a known destination on the resistance surface, the connectivity predictions of Circuitscape typically correlated more strongly than those of resistant kernels. Additionally, in this case the error from these two models roughly doubled, and the spatial overlap in the predictions from Circuitscape increased but still remained less than those from resistant kernels.

Importantly, we note here that in many applications of connectivity modelling, knowledge of the precise destination of movement is often very difficult to obtain, particularly with dispersing animals. This limits the effectiveness of models such as Circuitscape which require knowledge of a location, or region, towards which the movement is inclined. In contrast, this is a strength of resistant kernels modelling, which does not require the knowledge (or assumption) of a destination prior to movement.

### Connectivity modelling: limitations and future developments

The widespread usage and influence of the three models analysed in this study reflects the importance and centrality given to connectivity modelling in modern conservation science. Although such methods have grown in sophistication since the first least-cost path algorithms used in ecology two decades ago, there remain significant limitations to methods such as Circuitscape, factorial least-cost paths and resistant kernels, in their ability to understand and predict the pathways central to animal movement. For example, these models do not account for autocorrelation, spatial scale of movement, or mortality risk, despite the well-established and fundamental importance these factors have in shaping movement patterns^5,35–37^. Furthermore, as noted in^21^, Circuitscape assumes a directional symmetry of resistance between adjacent pixels, despite movement from an area of higher to lower resistance being much more likely than the reverse.

Before the emergence of landscape resistance as a framework for mapping connectivity, some agent-based models did incorporate more detailed aspects of individual movement choice^38,39^, but the lack of spatial complexity in these earlier methods greatly limited their effective application to conservation science. Pathwalker, and other recent agent-based approaches like HexSim and RangeShifter^40,41^, combine the spatially-explicit approach of resistance-based algorithms with the detail and flexibility given to simulating movement behaviour enabled by agent-based modelling. Because landscape connectivity is a phenomenon *which arises from the cumulative movement routes of individual organisms*, these individual-based methods thus address some important drivers of connectivity which are missing in the simplistic assumptions used by Circuitscape, resistant kernels and factorial least-cost paths.

However, many fundamental drivers of movement are still left unaccounted for: resistance-based connectivity models assume landscape resistance and animal movement behaviour to remain unchanging as the animal moves through space and time, and thus they ignore spatiotemporal variation and other key contextual details. These factors often lie at the heart of shaping movement and connectivity. For example, seasonal variability in the landscape can substantially alter movement patterns^42,43^, and spatial variability in movement behaviour can result in radically different estimates of landscape resistance and connectivity^44,45^. Recent work in connectivity modelling, such as dynamic landscape connectivity^46^, has developed in response to these spatiotemporal limitations of resistance surfaces.

Moreover, the earth’s ecosystems are now increasingly shaped by anthropogenic presence. Since ecological processes in reality do not follow a neat distinction between ‘nature’ and ‘society’^7,47,48^, traditional movement models which ignore the complexities of human influence on animal behaviour for the sake of theoretical and algorithmic simplicity are growing increasingly unreliable^49–51^. Emerging studies empirically demonstrate the varied effects of human presence on movement patterns and their potentially substantial impact on connectivity predictions^52–54^. Similarly, the dynamic presence of other nonhuman animals can be a major force in shaping movement behaviour and the resulting connectivity pathways, particularly with dispersing individuals^55^.

All scientific models ostensibly require a degree of simplification of ecological dynamics. But many recent studies point towards the enormous effect that spatiotemporal variation and context dependence have on connectivity predictions, and the central importance for their inclusion into connectivity modelling. As demonstrated in the above literature, these key contextual details pose a particular challenge to the minimalist assumptions used in resistance surfaces and the major resistance-based connectivity models. Namely, the assumption that animals are automatons moving through a static landscape, according to a simplistic set of cost-benefit movement rules, reduces the rich dynamics of both animal and landscape to an extent that often renders such models ineffective for application to conservation practice and policy.

Despite the complexities involved in developing connectivity models which attend to spatiotemporal variation, interactions, and other important contextual details, emerging technologies and methodologies are making this integration increasingly possible^15^. For example, flexible spatially-explicit individual-based models like Pathwalker provide a powerful framework with which to explore new movement parameters and modelling techniques, using simulation to test a range of hypotheses concerning drivers of movement which may be difficult to investigate empirically. Moreover, since conservation science and landscape ecology are inherently interdisciplinary^56^, this is an excellent opportunity to draw upon adjacent developments in the conservation social sciences^57^. Doing so will likely enable the identification of new objectives and parameters for models to fit better with the realities of conservation practice^58–63^. Scientists and modellers who are willing to engage with this interdisciplinary literature may find themselves at the forefront of revolutionary advances in applied ecological research.

### Conclusion

The results of our study show that the choice of connectivity model, along with the degree of destination bias in the movement and spatial complexity of the landscape, had the greatest impact on the accuracy of connectivity predictions. Comparatively, aspects of movement behaviour such as autocorrelation and the spatial scale of response to the landscape had relatively little overall effect. We found the factorial least-cost path algorithm to be substantially less accurate than Circuitscape and resistant kernels across nearly all scenarios. Of these latter two models, resistant kernels was overall more accurate when movement was not inclined towards a particular known destination, and Circuitscape generally more accurate when there was destination bias. In practice however, knowledge of precise destinations are usually very difficult to obtain; connectivity models such as resistant kernels are thus likely to be more appropriate in a wider range of contexts.

Despite their widespread usage in conservation science, all three models are limited in their effective application and predictive abilities by their simplistic assumptions of animal movement. Many fundamental drivers of connectivity, such as spatiotemporal variability and the effects of human and nonhuman presence, are absent in the framework of landscape resistance and these resistance-based models. As discussed above, this is an important moment in the development of connectivity modelling: emerging methods and technologies are enabling us to move beyond the reductionist framework of landscape resistance as the primary basis for mapping connectivity. Recent spatially-explicit individual-based models, such as Pathwalker, provide a rich set of tools with which to explore and test a wide range of hypotheses concerning central questions in the field of landscape connectivity. Adjacent developments in the conservation social sciences provide an excellent range of methodologies and techniques for identifying new objectives and parameters in connectivity modelling. We sense this to be a tremendous and exciting opportunity for cross-disciplinary thought and collaboration, to create and develop connectivity models which better reflect the lively patterns and complexities of animal movement in our more-than-human world.

## Supplementary Information

Supplementary Information.

## Data Availability

Access to the datasets used in this study may be supplied by the corresponding author upon request.
