# Supplementary material for: Connectivity modelling in conservation science: a comparative evaluation
Source: Sci Rep. 2022 Oct 6;12:16680. doi: 10.1038/s41598-022-20370-w (PMC9537442; doi:10.1038/s41598-022-20370-w)
Supplement: Supplementary file 1 — Supplementary Information. [file 41598_2022_20370_MOESM1_ESM.pdf]

Supplementary figures and tables

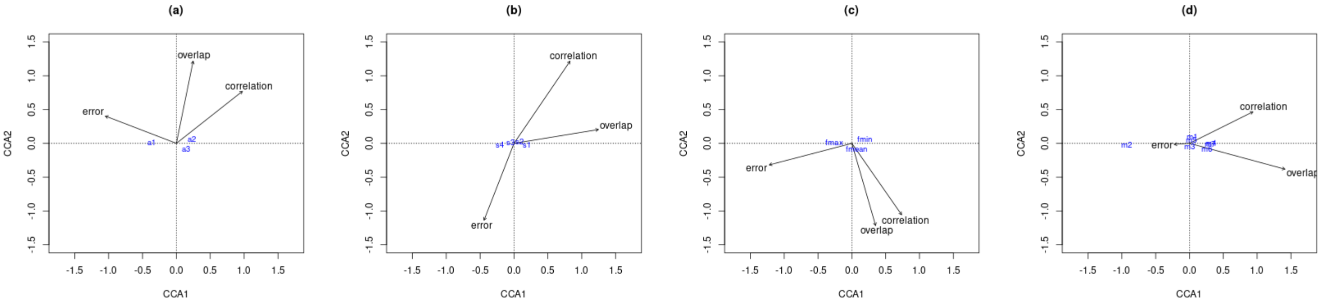

Figure S1: Additional CCA diagrams: (a-c) are from the first study, (d) is from the second study. A key to the CCA figure labels is provided in Supplementary Table S2. (a) shows that higher degrees of autocorrelated movement resulted in lower model error, but had little effect on correlation and overlap. Variation in spatial scale (b) and scale response function (c) had little overall effect on accuracy. However, the relatively low impact of spatial scale seen in (b) may reflect the small range of movement scales used in the simulation study; larger spatial scales of movement behaviour may result in a more substantial effect on model accuracy. The spread of points in (d) is similar to that in Figure 5(d), suggesting the effects of movement mechanism on model accuracy to be fairly consistent over different degrees of destination bias.

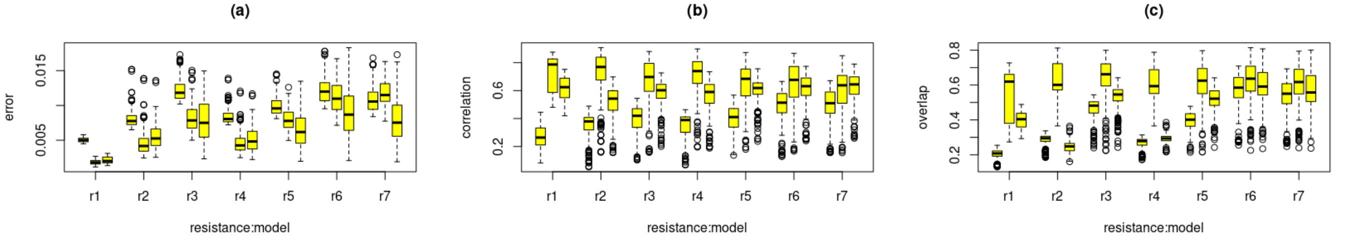

Figure S2: Boxplots illustrating the effects of landscape structure on model accuracy from the first study, displaying the error (a), correlation (b) and overlap (c). Each triplet of boxes represents factorial least-cost paths, resistant kernels and Circuitscape, from left to right. Across all three statistics, the three models perform more similarly as spatial complexity increases. For the simpler surfaces (r1, r2 and r4), resistant kernels is substantially more accurate than factorial least-cost paths and Circuitscape. For the more complex surfaces (r3, r6 and r7), error increases for all three models. However, the correlation and overlap for factorial least-cost paths and Circuitscape also increase, while resistant kernels remain consistent with high correlation and overlap.

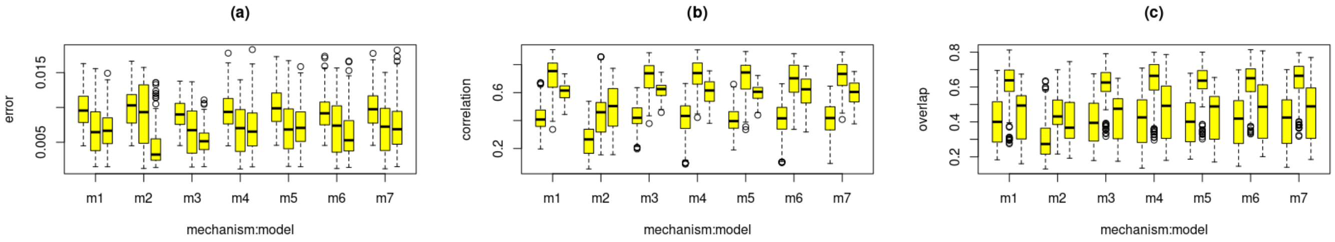

Figure S3: Boxplots illustrating the effects of each movement mechanism on model accuracy from the first study, displaying the error (a), correlation (b) and overlap (c). Each triplet of boxes represents factorial least-cost paths, resistant kernels and Circuitscape, from left to right. Aside from the attraction mechanism without energy or risk (m2), each model maintains fairly consistent accuracy across the movement mechanisms: resistant kernels has substantially higher correlation and overlap than Circuitscape, and these two models produce predictions with similar levels of error; factorial least-cost paths is the least accurate in every case. With the attraction mechanism, in the absence of energy or risk, Circuitscape has much lower error and similar levels of correlation and overlap to resistant kernels.

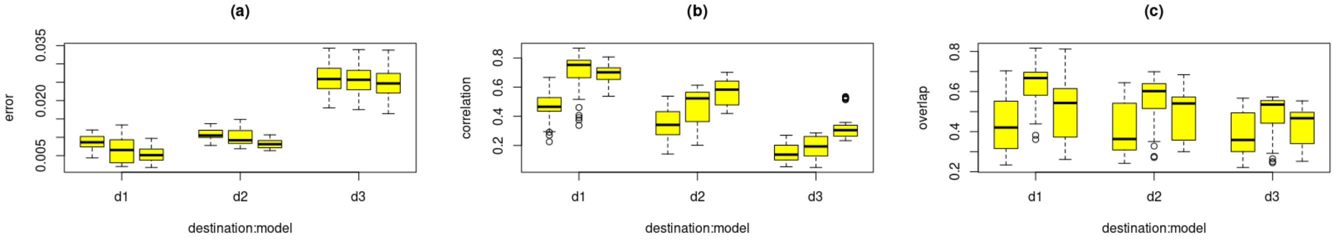

Figure S4: Boxplots illustrating the interaction between model accuracy and the degree of destination bias from the second study, displaying the error (a), correlation (b) and overlap (c). Each triplet of boxes represents factorial least-cost paths, resistant kernels and Circuitscape, from left to right. (a) shows Circuitscape to produce predictions with the lowest error across all levels of destination bias, although the difference between the three models is only marginal. (b) demonstrates that, at low levels of destination bias, predictions by resistant kernels correlate most strongly with the simulated connectivity maps; for higher degrees, CircuitScape gives predictions with greatest linear correlation. In (c), we see resistant kernels to consistently result in the highest spatial overlap.

Parameter	DF	SS	MS	F-value	P-value
Mechanism	1	2.30	2.299	111.808	<2e-16
Resistance	1	10.54	10.539	512.602	<2e-16
Scale	1	1.11	1.115	54.220	2.07e-13
Model	1	19.07	19.073	927.675	<2e-16
Mechanism:Resistance	1	0.46	0.460	22.390	2.28e-06
Mechanism:Scale	1	0.00	0.000	0.005	0.94554
Mechanism:Model	1	0.01	0.007	0.359	0.54924
Resistance:Scale	1	0.15	0.147	7.170	0.00744
Resistance:Model	1	3.49	3.490	169.726	<2e-16
Scale:Model	1	0.11	0.108	5.237	0.02215
Residuals	5281	108.58	0.021		

Table S1: Four-way analysis of variance table for movement mechanism, resistance surface, spatial scale and model choice. Strong interaction is seen between model choice and resistance surface, suggesting the three models to differ in effectiveness as spatial complexity varies. Mechanism and resistance surface also show substantial interaction.

Parameter	Label	Value
Model	flcp	factorial least-cost paths
	rk	resistant kernels
	cs	Circuitscape
Resistance surface	r1	resistance surface 1
	r2	resistance surface 2
	r3	resistance surface 3
	r4	resistance surface 4
	r5	resistance surface 5
	r6	resistance surface 6
	r7	resistance surface 7
Mechanism	m1	energy
	m2	attraction
	m3	risk
	m4	energy + attraction
	m5	energy + risk
	m6	attraction + risk
	m7	energy + attraction + risk
Autocorrelation	a1	0
	a2	0.35
	a3	0.75
Spatial scale	s1	1x1
	s2	3x3
	s3	5x5
	s4	7x7
Scaling function	fmean	focal mean
	fmax	focal maximum
	fmin	focal minimum
Destination bias	d1	0.1
	d2	0.3
	d3	0.6

Table S2: Key to figure labels for diagrams in main text and supplementary material.
